# Comparison of Tooth Discoloration Induced by Mineral Trioxide Aggregate and Polycarboxylate Cement Containing Potassium Nitrate in the Presence and Absence of Blood: An Ex Vivo Study

**DOI:** 10.1155/ijod/1663827

**Published:** 2026-10-12

**Authors:** Alireza Adl, Fereshte Sobhnamayan, Maziyar Ghani Kolahloo

**Affiliations:** ^1^ Department of Endodontics Biomaterials Research Center School of Dentistry Shiraz University of Medical Sciences Shiraz Iran sums.ac.ir; ^2^ Department of Endodontics School of Dentistry Shiraz University of Medical Sciences Shiraz Iran sums.ac.ir; ^3^ Student Research Committee School of Dentistry Shiraz University of Medical Sciences Shiraz Iran sums.ac.ir

**Keywords:** mineral trioxide aggregate, polycarboxylate cement, potassium nitrate, pulpotomy, tooth discoloration

## Abstract

**Purpose:**

Most materials used in endodontic procedures may cause tooth discoloration. This study aimed to compare coronal tooth discoloration after application of mineral trioxide aggregate (MTA) and polycarboxylate cement containing 5 wt% potassium nitrate (PCPN) as vital pulp therapy materials in the presence and absence of blood.

**Methods:**

Sixty‐eight extracted human single‐rooted anterior and premolar teeth were selected, prepared, and standardized to 5 mm root length. After access cavity preparation and enlargement of canals up to Glidden drills #6, cylindrical pieces of white sponge were placed into the canals up to the cementoenamel junction. Specimens were then randomly assigned into two experimental groups (*n* = 30 each) in which the sponge was saturated with blood or saline solution. In each group, the pulp chambers were filled with 3‐mm thickness of MTA or PCPN (*n* = 15 each) and cavities were sealed with composite after 24 h. Eight additional teeth (four in each of the blood and saline groups) were prepared without placement of any pulp capping material. All samples were incubated at 37°C and 100% humidity. Color changes (Δ*E*) were measured using a spectrophotometer at baseline, 1 day, 1 month, 3 months, and 6 months. Data were analyzed using repeated measures ANOVA, one‐way ANOVA, Tukey post hoc, and paired *t*‐tests (*α* = 0.05).

**Results:**

A significant interaction was observed between time and groups (*p* < 0.001). In the absence of blood, there was no significant difference in discoloration between MTA and PCPN. In the presence of blood, MTA showed greater discoloration at 3 and 6 months compared with PCPN (*p* < 0.05). Tooth color change in both experimental groups increased after 1 month (*p* < 0.05).

**Conclusions:**

MTA exhibited more severe tooth discoloration than PCPN when used in the presence of blood. Both materials showed increased discoloration after 1 month.

## 1. Introduction

Tooth discoloration is one of the major esthetic concerns for both patients and dentists, especially when the anterior teeth are involved [1]. Tooth discoloration can have a significant impact on an individual’s quality of life [2]. Many materials used in endodontic treatments may lead to tooth discoloration and an undesirable outcome [1].

Managing pulpal injuries in teeth with immature root development presents major clinical challenges. Teeth with open apices and pulpal involvement can be treated with vital pulp therapy, including direct pulp capping or pulpotomy. In the latter, part of the coronal pulp tissue is removed, and after controlling the bleeding, a biomaterial is placed over the pulp to allow the root to continue its development and maturation [3].

Various materials have been used for pulp coverage in vital pulp therapy procedures, but in recent years, mineral trioxide aggregate (MTA) has been widely used [4]. MTA was introduced to endodontics in 1993 by Torabinejad and colleagues. Its major components include tricalcium silicate, tricalcium aluminate, tricalcium oxide, and silicate oxide [5].

Previous studies have demonstrated that MTA possesses favorable physical properties, such as excellent sealing ability [6] and marginal adaptation [7]. Furthermore, due to its long‐term high pH, MTA can eliminate bacteria and create a suitable environment for tissue repair [8].

In addition, laboratory studies have shown that MTA does not induce apoptosis in pulp cells and actually promotes pulp cell proliferation [9]. Clinical studies have also reported high success rates for pulpotomy treatments using this material [10, 11].

Despite these advantages, MTA has several drawbacks, including difficult handling, long setting time, high cost, and the potential for tooth discoloration [5, 12, 13].

Initially, MTA was introduced in its gray form (GMTA), which caused pronounced crown discoloration [14, 15]. Consequently, a white form (WMTA) was developed by modifying its chemical composition. In white MTA, the amount of certain metallic oxides—such as Fe_2_O_3_, Bi_2_O_3_, Al_2_O_3_, and MgO—responsible for discoloration was reduced [16]. However, several studies have demonstrated that even white MTA can cause crown discoloration, particularly when it is used in the presence of blood. In an ex vivo study, Shokouhinejad et al. [17] compared tooth discoloration following the application of ProRoot MTA, Biodentine, Ortho MTA, and ERRM in the presence and absence of blood. They found that tooth color change increased over time in all experimental groups and that blood contamination significantly increased tooth discoloration. However, no significant difference was observed among the four materials.

Another study evaluated tooth discoloration following treatment with MTA Angelus, calcium‐enriched mixture (CEM) cement, and Biodentine. The results demonstrated that discoloration increased over time and that the presence of blood was associated with greater discoloration. Moreover, discoloration caused by Biodentine in both the presence and absence of blood was significantly lower than that caused by MTA, whereas no significant difference was observed between Biodentine and CEM cement [18].

In another study, Palma et al. [19] assessed and compared coronal discoloration induced by four endodontic biomaterials used in regenerative endodontic procedures in the presence and absence of blood. Their findings suggested that, after 6 months of evaluation, blood exposure might be a critical factor influencing biomaterial‐related color changes. Biodentine showed the lowest discoloration potential, followed by TotalFill and PCM, with no statistically significant differences among these materials. MTA exhibited the greatest color variation, resulting in a noticeable increase in tooth discoloration over time.

More recently, Uslu et al. [20] evaluated whether the application of a dentin bonding agent or Nd:YAG laser irradiation could reduce tooth discoloration induced by different calcium silicate‐based cements (CSCs) under blood‐contaminated conditions simulating regenerative endodontic procedures. Their findings showed that neither dentin bonding agent application nor Nd:YAG laser pretreatment completely prevented CSC‐related discoloration. ProRoot MTA showed the greatest discoloration potential, even after preventive dentin pretreatment.

A combination of polycarboxylate cement containing 5% potassium nitrate (PCPN) has been previously recommended for preserving pulp vitality in direct pulp capping [21, 22]. A clinical study showed that when this combination is used as a pulp capping material in deep caries, the 4‐year success rate is 98% [21]. Moreover, clinically, PCPN was reported to eliminate or decrease postoperative pain significantly [22]. It seems that potassium nitrate depolarizes the nerve fibers, and the dentinal‐pulpal circulation and natural defense system remain functionally intact and are even improved because nitrate salts tend to increase circulation by being converted to nitric oxide [23].

In a study on dogs, the application of PCPN for direct capping of traumatically exposed pulp did not result in any degenerative changes in the dental pulp. The authors attributed the results to the good adhesive properties of PCPN that guaranteed the closure of the cavity and good and lasting protection of traumatically exposed dental pulp, creating optimal conditions for expression of its natural reparative potential [24].

In a 2021 clinical trial, Ahmed et al. compared PCPN with MTA in pulpotomy of immature mandibular first molars. The results after 12 months showed a 92% success rate in both groups, defined by the absence of clinical symptoms and continued root development. Based on these findings, as well as its lower cost and easier handling, the authors suggested that PCPN could serve as an alternative biomaterial to MTA [25].

Sayed et al. [26] compared the effects of PCPN and MTA for direct pulp capping in immature dog teeth. They reported no evidence of inflammatory reactions in either group throughout the 3‐month observation period. Hard tissue formation was observed in both groups; however, the extent of hard tissue formation was greater in the MTA group than in the PCPN group.

In 2024, Elheeny et al. [27] compared the clinical and radiographic success of PCPN to that of MTA in the direct pulp cap of carious, immature permanent molars. Their results showed a similar success rate after 18 months of follow‐up between the two materials. In addition, root maturation indicators showed no significant difference between the two groups over the follow‐up period. In 2025, in the study by Dahlawi et al. [28], PCPN demonstrated a balanced profile of immunomodulation, regenerative signaling, and mechanical integrity, supporting its potential use in vital pulp therapy. Moreover, in a 2026 study, PCPM exhibited significantly higher shear bond strength to composite resin than MTA and also showed potential for immediate restoration in vital pulp therapy [29].

Since one of the main drawbacks of using MTA in pulpotomy is tooth discoloration—and no studies have yet evaluated the discoloration potential of PCPM, the aim of the present study was to compare the tooth discoloration effects of these two materials in the presence and absence of blood. The null hypothesis was that there would be no significant difference in tooth discoloration between MTA and PCPN in the presence and absence of blood contamination.

### 1.1. Sample Size Determination

The sample size was determined based on the study by Valles et al. [30]. The calculation was based on a difference in mean values of 8.89 between the two groups reported in that study, with standard deviations of 3.15 and 1.75 for Groups 1 and 2, respectively. Assuming a two‐sided type I error (*α*) of 0.05 and a statistical power of 90%, the minimum required sample size was calculated to be 3 specimens per group. To allow for comparisons under both the presence and absence of blood, as well as subgroup comparisons, 15 specimens were included in each experimental subgroup.

## 2. Methods and Materials

This study was approved by the Ethics Committee of Shiraz University of Medical Sciences under the ethical code IR.SUMS.DENTAL.REC.1402.097. Our methodology was adopted from the study by Shokouhinejad et al. [17] and modified to suit our experimental requirements.

A total of 68 human single‐rooted permanent anterior and premolar teeth extracted for periodontal or orthodontic reasons were selected for this study. Informed consent was routinely obtained from the patients or their legal guardians for the use of extracted teeth for research purposes. The study was conducted in accordance with the Declaration of Helsinki.

The teeth were fully formed and were examined visually and radiographically to confirm the absence of cracks, fractures, caries, calcified canals, previous root canal treatment, resorption, and coronal restoration [31]. The reason for tooth extraction was unrelated to this study, and neither the dentist who prescribed the extraction nor the person who performed it had any involvement in this research.

Initially, the teeth were disinfected in a 2.5% sodium hypochlorite solution for 1 h and then stored in normal saline [17]. The maximum storage period between tooth extraction and the initiation of the experiment was limited to 1 month. This relatively short and standardized storage period was used to minimize potential changes in the physical and optical properties of the extracted teeth during storage.

The teeth were cleansed with an ultrasonic scaler and polished with pumice and water to remove extrinsic debris and stains. To standardize the samples, the apical portion of each root was sectioned perpendicular to the long axis using a diamond bur, leaving 5 mm of the root intact.

Standard access cavities were prepared, and the root canals were cleaned and shaped using Gates‐Glidden drills sizes 1–6 (MANI GATES, Utsunomiya, Tochigi, Japan). After each instrument, the canals were irrigated with 2 mL of 2.5% sodium hypochlorite. Upon completion of instrumentation, 5 mL of 17% EDTA (CERKAMED, Poland) was applied for 1 min, followed by 10 mL of normal saline as the final rinse.

After drying the canals, a customized white cylindrical sponge was placed from the apical end up to the cementoenamel junction of each sample. The apical end and dentin walls were then etched, bonded, and sealed with A3 shade composite resin (Aelite All‐Purpose Body, Bisco, USA) as an apical restoration.

At this stage, all 68 teeth were placed in a single container and thoroughly mixed. Thirty teeth were randomly selected and assigned to the blood group, and another 30 teeth were randomly selected and assigned to the normal saline group. The remaining eight teeth were randomly allocated into two groups of four and assigned to either the blood or normal saline condition without receiving any pulpotomy material. The 30 teeth in each of the blood and normal saline groups were then randomly subdivided into two experimental subgroups (*n* = 15) according to the pulpotomy material used: MTA (Ortho MTA, BioMTA Co., South Korea) or PCPC. Fresh human blood was collected by a trained individual from a healthy volunteer using a syringe containing EDTA to prevent clotting. The sponges of the teeth assigned to the blood groups were saturated with fresh blood, whereas those assigned to the normal saline groups were saturated with normal saline using an insulin syringe. Care was taken to avoid contamination of the access cavity walls.

The PCPN mixture was prepared using a digital scale (AND GR‐300, Higashi‐Ikebukuro, Toshima‐ku, Tokyo, Japan) by mixing 95 wt% polycarboxylate cement powder (Master‐Dent, Dentonics Inc., Monroe, NC, USA) with 5 wt% potassium nitrate powder (NP1K, Falatkala, Zanjan, Iran).

MTA and PCPN pastes were prepared according to the manufacturer’s instructions, and a 3 mm thickness of each was placed inside the access cavity using an MTA gun (Medesy, Maniago, Italy), and a flat plastic instrument was used with minimal pressure to ensure contact with the coronal surface of the sponge. After placement of either MTA or PCPC, the pulp chamber walls were gently cleaned using a sterile cotton pellet moistened with sterile normal saline to remove any residual material from the chamber walls.

A moistened cotton pellet was placed over each material to allow setting. After 24 h, all access cavities were restored with a microhybrid composite resin (Filtek Z250, SHADE a3, 3M ESPE, USA). The samples were then stored in an incubator at 37°C and 100% humidity.

The remaining eight teeth were divided into two groups of blood or normal saline (*n* = 4) and underwent the same procedures except that no material was placed in the pulp chamber; the access cavities were completely restored with A3 composite resin.

### 2.1. Color Measurement

For color evaluation using a spectrophotometer (VITA, Bad Säckingen, Germany), each sample was embedded in a putty mold. A hole was created on the buccal surface of the putty at the interface of the cervical and middle thirds of the crowns, corresponding to the detector diameter of the spectrophotometer to ensure consistent measurement points.

The spectrophotometer was calibrated according to the manufacturer’s instructions before each measurement session. All measurements were performed in the same room under consistent lighting conditions, with the specimens positioned in the same orientation and at the same measurement site throughout the experimental period.

Color change was evaluated at five intervals: after access cavity preparation (*t*_0_), 1 day (*t*_1_), 1 month (*t*_2_), 3 months (*t*_3_), and 6 months (*t*_4_) after material placement under identical lighting conditions. A single operator, who was blinded to group allocation, performed all spectrophotometric measurements. Each specimen was measured three times, and the mean of the three measurements was used for statistical analysis. Data were reported using the Commission International de I’Eclairage’s *L*  ^∗^ *a*  ^∗^ *b*  ^∗^ color system, and the color change between two measurements was calculated using the following formula:

ΔE∗=L1−L0∗+a1−a0∗+b1−b0∗212.

*L*  ^∗^ values represent lightness, ranging from black (0) to white (100), and *a*  ^∗^ and *b*  ^∗^ represent greenness/redness and blueness/yellowness, respectively.

### 2.2. Statistical Analysis

Data were analyzed using repeated‐measures ANOVA to assess the effects of material type, blood contamination, and time on tooth discoloration (Δ*E*). Given the significant time × group interaction, one‐way ANOVA followed by Tukey’s post hoc test was performed for intergroup comparisons at each time point. For intragroup comparisons of each material over time, paired *t*‐tests with Bonferroni correction were applied. A *p* value of <0.05 was considered statistically significant for all analyses.

## 3. Results

The mean and standard deviation of color change (Δ*E*) values for all experimental groups at different time intervals are presented in Table 1 and Figure 1.

**Figure 1:**
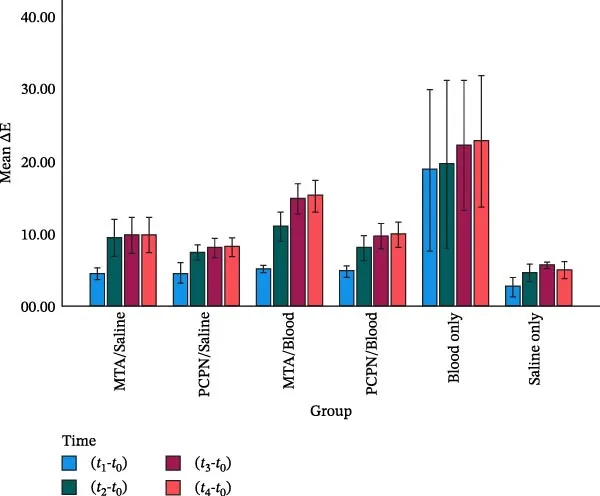
Graph of the mean and standard deviation (Δ*E*) of different groups at different time intervals.

**Table 1:** Mean and standard deviation (Δ*E*) of different groups at different time intervals.

Group	Δ*E*_1_ (*t*_1_–*t*_0_)	Δ*E*_2_ (*t*_2_–*t*_0_)	Δ*E*_3_ (*t*_3_–*t*_0_)	Δ*E*_4_ (*t*_4_–*t*_0_)
MTA/saline	Aa	ABb	Ab	Ab
	4.47 (1.47)	9.39 (4.6)	9.74 (4.49)	9.79 (4.4)
PCPN/saline	Aa	Ab	Ab	Ab
	4.59 (2.54)	7.38 (1.89)	8.01 (2.33)	8.10 (2.31)
MTA/Blood	Aa	Bb	Bc	Bc
	5.1 (0.89)	10.91 (3.05)	14.75 (3.71)	15.14 (3.92)
PCPN/Blood	Aa	ABb	Ab	Ab
	4.75 (1.39)	7.96 (3.05)	9.062 (3.18)	9.79 (3.1)

*Note*: Different capital letters indicate statistical differences in column comparisons, and different lowercase letters indicate statistical differences in row comparisons.

A significant interaction was observed between time and groups (*p* < 0.001) (Figure 2).

**Figure 2:**
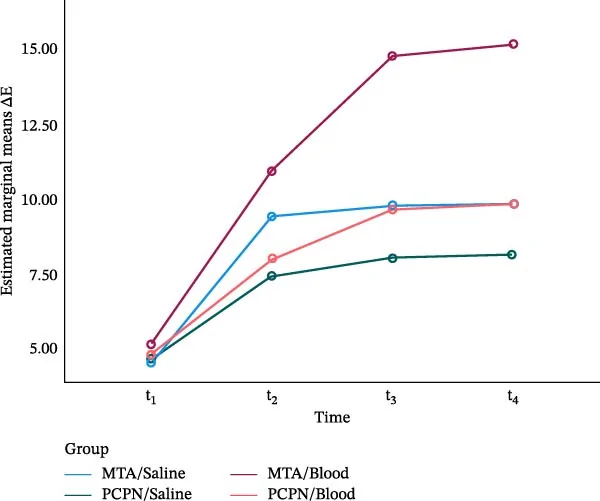
Color change trend in the groups under study.

At the 1‐day interval, there was no significant difference in color change among the groups (*p* = 0.75).

However, at the 1‐, 3‐, and 6‐month intervals, the MTA/Blood group exhibited the greatest discoloration. At 1 month, this group showed a significant difference only with the PCPN/Saline group (*p* < 0.01), while at 3 and 6 months, it demonstrated significantly higher discoloration than all other groups (*p* < 0.05). No significant differences were found among the remaining groups (*p* > 0.05). Comparison of each experimental group over different time intervals revealed that, in all groups, the Δ*E* value at 1 month was significantly higher than at the first day (*p* < 0.001). In the PCPN/Saline, MTA/Saline, and PCPN/Blood groups, no significant differences were observed in color change between the 1‐, 3‐, and 6‐month intervals (*p* > 0.008). However, in the MTA/Blood group, the Δ*E* value after 3 months was significantly greater than that at 1 month (*p* = 0.001).

## 4. Discussion

The aim of this ex vivo study was to compare the degree of coronal tooth discoloration following the application of MTA and PCPN as vital pulp therapy materials in the presence and absence of blood. Extracted human teeth were used in this study. After preparing the access cavities and placing the test materials, the cavities were restored with a resin composite.

It should be noted that in some previous studies evaluating discoloration caused by vital pulp therapy materials, a closed system was used—meaning that the access cavity was not prepared, and the tested materials were introduced into the pulp chamber through the apical end of the root [32, 33]. Clearly, such a model does not simulate clinical conditions as complete removal of pulpal remnants from the pulp chamber and pulp horns is difficult [17]. Moreover, Marciano et al. [12] reported that composite restoration does not play a role in the discoloration induced by CSCs.

In the present study, fresh human blood was used to evaluate the influence of blood contamination on tooth discoloration following the application of the two pulpotomy materials. The experimental model used here closely simulated the clinical situation of vital pulp therapy and allowed the reproduction of coronal discoloration in the presence of blood.

In this study, color evaluation was performed using a spectrophotometer and the CIELAB color system, which is widely used in dental research because of its standardized, objective, and reproducible representation of color in terms of lightness (*L*  ^∗^), red‐green (*a*  ^∗^), and yellow‐blue (*b*  ^∗^) coordinates. An important advantage of CIELAB is that changes in these individual color attributes can be evaluated separately, while its widespread use also facilitates comparison with previous studies on tooth discoloration.

Nevertheless, the limitations of the CIELAB color‐difference formula should be acknowledged when interpreting the clinical relevance of the present findings. The conventional Δ*E*_ab_ calculation represents the Euclidean distance between two points in the CIELAB space and does not always correspond closely to human visual perception across the entire color space. CIEDE2000 was developed to improve this perceptual agreement by incorporating corrections for differences in lightness, chroma, and hue. Previous studies in dentistry have reported that CIEDE2000 provides a better fit to visually perceived color differences than CIELAB in several dental applications. Therefore, although CIELAB remains useful and facilitates comparison with previous discoloration studies, CIEDE2000 may provide a more perceptually relevant approach for future investigations.

In this study, color evaluation was performed using a spectrophotometer and the CIE*L*  ^∗^ *a*  ^∗^ *b*  ^∗^ color system, as this method can detect even minimal color differences while offering high reproducibility, objectivity, and sensitivity [34]. Due to its precision and reliability, this system is considered an appropriate quantitative method for color evaluation [14]. Another important advantage of CIELAB is that changes in these individual color attributes can be evaluated separately, while its widespread use also facilitates comparison with previous studies on tooth discoloration.

Nevertheless, the limitations of the CIELAB color‐difference formula should be acknowledged when interpreting the clinical relevance of the present findings. The conventional Δ*E*_ab_ calculation represents the Euclidean distance between two points in the CIELAB space and does not always correspond closely to human visual perception across the entire color space. CIEDE2000 was developed to improve this perceptual agreement by incorporating corrections for differences in lightness, chroma, and hue. Previous studies in dentistry have reported that CIEDE2000 provides a better fit to visually perceived color differences than CIELAB in several dental applications. Therefore, although CIELAB remains useful and facilitates comparison with previous discoloration studies, CIEDE2000 may provide a more perceptually relevant approach for future investigations [35].

The findings of this study led to the rejection of the null hypothesis. A significant interaction between time and groups was observed, indicating that the pattern of discoloration in the experimental groups varied over time. The findings showed that in the absence of blood, there were no statistically significant differences between MTA and PCPN at any evaluated time point. However, the mean discoloration value of MTA was higher than that of PCPN from the first month onward. Although this difference was not statistically significant, it may still have clinical relevance in the long term.

In the presence of blood, MTA produced greater discoloration than PCPN at all time points, though this difference was statistically significant only at 3 and 6 months. Furthermore, no significant differences were observed between PCPN/saline and PCPN/Blood, suggesting that PCPN is not affected or is minimally affected by blood contamination. An additional factor that may explain the superior color stability of PCPN could be the relatively shorter setting time (7–9 min) of conventional polycarboxylate cements [36]. Given that these materials typically harden more rapidly compared with calcium‐silicate cements, the reduced exposure time to blood may limit the infiltration of red‐blood‐cell components and metallic ions. However, there are no published data that specifically report the exact setting time of the polycarboxylate cement when modified with potassium nitrate, nor is the influence of the potassium nitrate addition on setting kinetics and the resultant color change currently documented. Therefore, while this hypothesis is plausible, further investigation is required to substantiate this potential mechanism.

Opposite to PCPN, MTA/Blood exhibited significantly greater discoloration compared with MTA/saline at 3 and 6 months. These findings are consistent with those of Shokouhinejad et al. [17], who reported that blood contamination increased the Δ*E* value of various calcium silicate‐based cements, including Ortho MTA, Biodentine, and EndoSequence Root Repair Material. Similarly, Lenherr et al. [37] demonstrated that both white and gray ProRoot MTA and Portland cement exhibited greater discoloration after blood exposure. Felman and Parashos [32] also showed that the presence of blood increased discoloration in white ProRoot MTA.

The exact mechanism underlying the enhanced discoloration of calcium silicate cements in the presence of blood remains unclear. One possible explanation is the penetration of red blood cells into the tooth structure [38]. It has also been proposed that blood may become trapped within the porosities of MTA and similar materials, leading to discoloration [37].

In addition to blood contamination, the composition of the material itself plays an important role in its discoloration potential. The presence of metal ions such as bismuth, iron, aluminum, and manganese in MTA can contribute to the color change. For white MTA, a possible mechanism is the oxidation of iron compounds [32].

It should also be noted that the saline and blood groups without pulpotomy materials were not intended to serve as positive or negative control groups. Rather, they were included to evaluate the degree of tooth discoloration in the absence of any pulpotomy material. Accordingly, these groups were not included in the statistical analyses comparing the experimental groups. This approach was adopted based on the study by Shokouhinejad et al. [17], which similarly included four specimens in the saline group and four specimens in the blood group without calcium silicate‐based materials for this purpose.

As expected, the blood‐only group exhibited severe discoloration, while the saline group showed minimal color change. The mild discoloration observed in the saline group was also reported by Shokouhinejad et al. [17] and was attributed to the larger volume of composite resin used for restoring the access cavity compared to the groups in which part of the cavity was filled with pulp capping materials.

In the current study, all experimental groups showed a noticeable increase in discoloration after 1 month compared to 1 day. Over time, at the 3‐ and 6‐month intervals, all groups demonstrated further color change; however, this increase was statistically significant only for the MTA/Blood group, while the other groups (MTA/Saline, PCPN/Saline, and PCPN/Blood) showed no significant changes. This gradual increase in discoloration with time has also been reported in numerous studies on MTA [13, 17, 30, 39].

Beyond statistical significance, the clinical relevance of the observed color changes should also be considered. Based on the perceptibility and acceptability thresholds reported in the dental literature, the Δ*E* values observed in the present study exceeded commonly reported clinically relevant thresholds [40]. In particular, all experimental groups showed Δ*E* values greater than 3.7 at all evaluated time points, indicating that the observed discoloration was likely to be visually detectable and potentially clinically relevant [13]. However, because perceptibility and acceptability thresholds vary according to the color‐difference formula, material, experimental conditions, and observer characteristics, this threshold should be interpreted with caution. This finding may be related, at least in part, to the pulpotomy procedure itself, including changes in the optical properties and translucency of the tooth structure following access preparation and placement of the pulpotomy material, rather than being attributable solely to the specific material used.

Finally, it should be emphasized that this ex vivo study cannot fully replicate clinical conditions. The discoloration values reported here represent quantitative measurements obtained by spectrophotometric analysis, which do not necessarily correspond to clinically perceptible color changes. Therefore, further in vivo clinical studies are required to confirm and extend these findings. Another limitation of the present study was the inclusion of both anterior teeth and premolars. Although anterior teeth are generally considered more appropriate for in vitro discoloration studies because coronal discoloration in these teeth is more clinically relevant [31], the inclusion of premolars was necessary to obtain a sufficient number of intact, caries‐free teeth without resorption or other structural alteration. Nevertheless, differences in tooth morphology, crown dimensions, and optical characteristics between anterior teeth and premolars may have introduced some variability in the assessment of tooth discoloration.

The use of 2.5% sodium hypochlorite for disinfection represents a potential limitation of the present study. Although all specimens underwent the same NaOCl exposure, its oxidizing properties may have affected the optical characteristics of the dental tissues and, consequently, subsequent discoloration measurements. Because no specific neutralization step was performed to confirm the complete elimination of residual NaOCl activity, a potential effect on baseline tooth color and subsequent color changes cannot be completely excluded. Future studies should consider validated neutralization protocols or alternative disinfection methods to minimize this potential confounding effect.

Another limitation of the present study is that tooth discoloration was assessed exclusively using spectrophotometric measurements. Spectrophotometry is a well‐established, objective, and reproducible method for quantifying tooth color changes; however, it provides colorimetric measurements at predefined areas and may not fully capture the spatial distribution or visual pattern of discoloration. Recent evidence suggests that standardized digital image analysis may provide complementary information regarding the extent and distribution of tooth discoloration. Karadayi et al. [41] demonstrated that combining spectrophotometric measurements with digital image analysis can provide a more comprehensive assessment of tooth discoloration induced by bioceramic sealers. Therefore, the absence of digital image analysis should be acknowledged as a limitation of the present study. Future studies combining spectrophotometry with standardized digital image analysis may provide a more comprehensive assessment of both the magnitude and spatial characteristics of tooth discoloration.

Another limitation of the present study is the relatively short follow‐up period of 6 months. Although a 6‐month observation period is commonly used in previous in vitro discoloration studies, it may not adequately reflect longer‐term changes in tooth color. Therefore, future studies with longer follow‐up periods are warranted to determine whether the observed discoloration persists, progresses, or stabilizes over time.

## 5. Conclusion

Within the limitations of this study, it can be concluded that in the absence of blood, no significant difference exists between MTA and PCPN. However, in the presence of blood, MTA causes more severe discoloration. Both materials showed an increase in discoloration after 1 month compared to baseline.

**Nomenclature**
MTA: Mineral trioxide aggregate
PCPN: Polycarboxylate cement containing 5 wt% potassium nitrate.

## Funding

The study has no funds.

## Ethics Statement

Ethical approval was granted for this project by the Ethics Committee of Shiraz University of Medical Sciences (IR.SUMS.DENTAL.REC.1402.097). The samples were accessed for research purposes on December 28, 2023. All data were fully anonymized before accessing, and the subject’s consent was received for the processing.

## Conflicts of Interest

The authors declare no conflicts of interest.

## Data Availability

The data are available in case of sending an official request letter.
